# Health Utilities of Bilateral Severe-to-Profound Hearing Loss with Assistive Devices

**DOI:** 10.3390/healthcare11111649

**Published:** 2023-06-05

**Authors:** Yi-Wen Chen, Pei-Hsuan Lin, Te-Yung Fang, Chen-Chi Wu, Pa-Chun Wang, Han Wang, Yu Ko

**Affiliations:** 1 Department of Pharmacy, Wan Fang Hospital, Taipei Medical University, Taipei 11696, Taiwan; 2 Department of Clinical Pharmacy, School of Pharmacy, College of Pharmacy, Taipei Medical University, Taipei 11031, Taiwan; 3 Department of Otolaryngology, National Taiwan University Hospital, Taipei 100225, Taiwan; 4 Department of Otolaryngology, Cathay General Hospital, Taipei 10630, Taiwan; 5 School of Medicine, Fu-Jen Catholic University, New Taipei City 24205, Taiwan; 6 Department of Medical Research, National Taiwan University Hospital, Hsin-Chu Branch, Hsinchu 30261, Taiwan; 7 Hearing and Speech Center, National Taiwan University Hospital, Taipei 100225, Taiwan; 8 Research Center for Pharmacoeconomics, College of Pharmacy, Taipei Medical University, Taipei 11031, Taiwan

**Keywords:** health utility, cochlear implants, hearing impairment

## Abstract

Hearing loss is a common sensory disorder in newborns. Early intervention with assistive devices benefits children’s auditory and speech performance. This study aimed to measure the health utilities of children with bilateral severe-to-profound hearing impairment with different assistive devices. The descriptions of four hypothetical health states were developed, and their utility values were obtained from healthcare professionals via the visual analogue scale (VAS) and time trade-off (TTO) methods. Thirty-seven healthcare professionals completed the TTO interview and were included in the analysis. The mean utility scores obtained via VAS were 0.31 for no assistive devices, 0.41 for bilateral hearing aids, 0.63 for bimodal hearing, and 0.82 for bilateral cochlear implants. As for the utility scores obtained via TTO, mean values were 0.60, 0.69, 0.81, and 0.90, respectively. None of the four groups had the same VAS- or TTO-elicited utility (*p* < 0.001). The post hoc test results showed that the difference was significant between any two groups (all *p* < 0.05). In conclusion, this study elicited health utility of bilateral hearing impairment with different assistive devices using the VAS and TTO methods. The utility values obtained provide critical data for future cost–utility analysis and health technology assessment.

## 1. Introduction

Hearing loss, which is the most common sensory disorder in newborns, occurs in between 1 and 3 of every 1000 newborns [1]. It results in difficulty receiving sound stimulations, which are critical to language and cognitive development. Consequently, hearing loss in children has a negative impact on learning and may also affect future employment prospects [2,3]. In view of its importance, there is consensus that early diagnosis of and intervention for childhood hearing loss are essential. Previous studies showed that early detection of hearing loss and intervention with assistive devices can improve auditory and speech performances, minimizing the developmental gap between hearing-impaired children and normal hearing peers [4,5]. As such, most developed and developing countries implemented universal newborn hearing screening for early identification of hearing impairment [6,7]. 

Among various hearing assistive devices, cochlear implant (CI) is of special concern from the socioeconomic perspective because of its relatively high cost and the need for a surgery to implant the device. Unlike hearing aids (HA), which work by amplifying sounds, CI is an implantable hearing device that directly transforms sounds into electrical impulses to stimulate functioning of the auditory nerve inside the cochlea. Accordingly, it brings benefits to people with severe-to-profound hearing loss who receive limited benefit from HAs. A CI consists of two parts: an external part and an internal part. The external part includes a microphone and a sound processor. The former works as the outer ear, which receives external sounds, while the processor transmits the sound signal to the subcutaneous signal receiver, i.e., the internal part. The receiver also plays the role of the basement membrane and hair cells. During the process, sound with different frequencies is decoded and separated. Finally, the signals stimulate the auditory nerves in different parts of the cochlea and are then interpreted by the brain. CIs can help patients with severe-to-profound sensorineural hearing loss, who have limited benefits from HAs, perceive the sensation of sound [8]. Positive outcomes with CIs were established in several studies. Children with CIs exhibit improved ability in hearing and communication, social interaction, and academic performance [9]. The benefits of cochlear implantation increase when the surgery is performed at a younger age and bilaterally. Children receiving early cochlear implantation usually reveal better language outcomes [5] and have higher opportunities to reach their normal age-equivalent developmental abilities and integrate into mainstream education [10]. 

Meanwhile, bilateral cochlear implantation provides advantages including head shadow effect, binaural summation, and binaural squelch. The process of sound identification relies on the collaborative efforts of both ears. Individuals with unilateral hearing loss face challenges in localizing sound sources since all voices appear to originate from their unaffected ear, making it difficult to determine the actual source. People with unilateral hearing loss often turn their heads and rely on visual cues to locate the origin of sounds. For people with binaural hearing, when a sound originates from a particular direction, the ear that is positioned closer to the sound source is known as the leading ear, while the ear further away is referred to as the lagging ear. The discrepancy in volume perceived by each ear provides us with vital information for localizing the source of the sound. This phenomenon is commonly referred to as the head shadow effect. In addition to the advantage of sound localization conferred by binaural hearing, there are two other benefits: binaural summation and binaural squelch. Binaural summation refers to the phenomenon wherein the brain perceives sounds to be louder when heard with both ears compared to a single ear, even if the volume of sound remains the same. This enhancement in perceived loudness can be attributed as the way in which sound information is processed by both hemispheres of the brain. Although sound initially enters each ear and is processed by the opposite hemisphere, ultimately both hemispheres work together to process and interpret the sound. The brain’s integration of sound information from both ears results in increased sensitivity to sounds, enabling clearer perception. Another advantage of binaural hearing is binaural squelch. In addition to amplifying sound by integrating information from both ears, binaural processing allows the brain to effectively focus auditory attention and filter out background noise. This process enables individuals to listen more effortlessly in noisy environments and concentrate on the speech of interest. Binaural squelch capitalizes on the brain’s ability to separate the desired signal from surrounding noise based on differences in the timing and intensity of sound received by each ear. This separation enhances the signal-to-noise ratio (SNR) and improves the clarity of the message being conveyed. Past studies showed that bilateral CIs provide better speech perception under noise and sound localization over unilateral implantation [11].

Pharmacoeconomics is the science of measuring costs and outcomes associated with the use of pharmaceuticals in healthcare services. Not limited to medication therapy, pharmacoeconomics can also be applied to other medical interventions, such as medical devices. Given finite resources, pharmacoeconomic studies are required to assist in decision-making in order to determine whether a drug or a medical device is cost-effective. These studies consider not only the direct costs of acquiring and administering medications or medical devices, but also their broader impact on overall healthcare expenditures and patient outcomes, such as quality of life. Health utilities are values that represent the strength of an individual’s preferences for specific health-related outcomes where higher values indicate better quality of life in those health states. The measurement of health utility is the key to calculating the health outcome measures in cost–utility analysis (CUA), which is a type of pharmacoeconomic evaluation that enables direct comparison between interventions in the same and different disease areas. Several studies measured health utilities for cochlear implantation [9,12]. A study conducted in Australia revealed that the utility of bilateral cochlear implantation was higher than those of unilateral implantation and pre-implantation status as valued via various health utility instruments, including time trade-off (TTO) [13]. In addition, a UK study reported similar results where health utility values were elicited via TTO [14]. TTO is a method used to measure a person’s preference for a certain health state. During the TTO procedures, respondents are asked to choose between two options: “living in a poor health state for X years” and “living in perfect health for a shorter time”. The response reflects the length of life that a person is willing to trade-off in order to regain perfect health and avoid staying in a poor health state [15]. The more unfavorable the health state to the respondent, the more time he/she is willing to sacrifice to avoid that particular health state. Health utility values can be elicited from patients, health professionals, and the general public to assess hypothetical or experienced health states. The utility values obtained can be used in CUA, which takes into account the clinical, economic, and quality of life outcomes in the evaluation of a health technology.

As the performance of CIs varies depending on language features [16], utility values derived from countries using different languages can also vary. To the best of our knowledge, few health utility assessments were previously conducted in Asia for hearing-related problems, let alone for cochlear implantation [17]. Several studies evaluated the effectiveness and cost-effectiveness of CI in Taiwan [18,19]. Considering the significant economic burden and quality of life impact associated with childhood hearing loss, more studies are required to evaluate the impact of different assistive devices in children with hearing loss. The utility values obtained in this study can contribute to future CUA and health technology assessments, providing important insights for decision-making and a reference for healthcare providers when determining an optimal treatment plan.

The present study aimed to use TTO to measure the health utilities of bilateral severe-to-profound hearing impairment with different assistive devices. To assess different devices’ impact on patients’ quality of life, four hypothetical health states were considered in the study: no assistive devices, bilateral HAs, bimodal hearing combines, and bilateral CIs.

## 2. Materials and Methods

**Figure 1:**
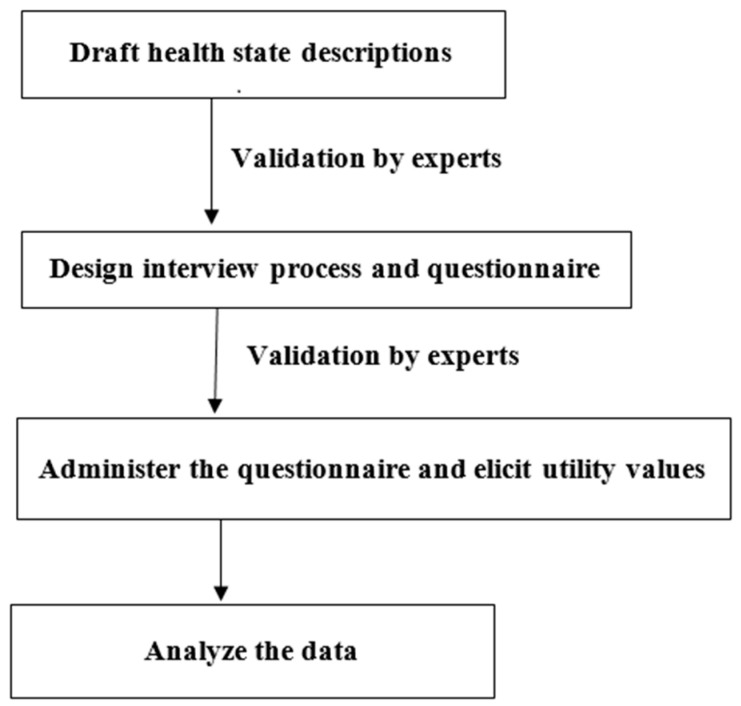
Flow chart of study design.

This study was a cross-sectional study using the visual analogue scale (VAS) and time trade-off (TTO) to measure the health utility values of four health states of interest. A flow chart of the study design is shown in Figure 1 and summarized in the below section. The initial step involved in this study was to define and establish a clear description for each health state, encompassing various dimensions of the daily experiences of children with severe hearing loss. These health states were described as hypothetical scenarios. To ensure the relevance of these scenarios to the daily lives of children with severe hearing loss, a process of description validation was conducted in individuals with expertise and experience in working with children with hearing loss. After the narratives of all the hypothetical scenarios were finalized, the researchers proceeded to conduct utility elicitation. The health states associated with the four hypothetical scenarios were referred to as scenarios 1–4. The interview process involved one-on-one interviews conducted either online via webcam or through face-to-face interactions, with VAS administered first, followed by TTO. During the interviews, the respondents were provided with the descriptions of all four health states. Through recalibrating the raw scores of the VAS and TTO, utility values were derived for each of the health states under consideration.

### 2.1. Development of Health State Descriptions

This study aimed to measure health utilities for children with bilateral severe-to-profound hearing loss with different assistive devices, including HA and CI, and four health states were selected for utility elicitation: without any assistive devices (None), bilateral hearing aids (HA/HA), bimodal hearing using a cochlear implant on one ear and a hearing aid on the other (HA/CI), and bilateral cochlear implants (CI/CI). To draft the descriptions of the four health states, the literature review was conducted to obtain information about the impact of hearing loss, as well as the impact of using assistive devices on various aspects of health in children with severe hearing loss [2,13,14,20,21]. These aspects included everyday sounds, communication patterns, social activities, speech development, safety issues, use of the telephone, future employment, and device-related concerns.

The drafted health state descriptions were then examined by experts who had experience taking care of children with bilateral hearing loss, including 11 parents, 5 otolaryngologists, 4 audiologists, 2 social workers, and 1 teacher from a special education school. The experts were asked to respond to three prompts for each narrative: (1) whether the narrative conformed to the real-life situation of hearing-impaired children; (2) whether and how the narrative could be amended to enhance clarity and comprehension; and (3) the important narratives or health aspects had been left out regarding hearing loss or assistive devices. Minor revisions of the descriptions were made by incorporating the experts’ feedback.

### 2.2. Health State Utility Measurement

#### 2.2.1. Participants

The participants in this study were recruited from three medical centers, three regional hospitals, and one hearing rehabilitation institute for children. Eligibility criteria were as follows: (1) being otolaryngologists, audiologists, or hearing-related healthcare professionals; (2) having CI-related working experience; and (3) having the ability to comprehend Chinese. The interviews were conducted from October 2021 to March 2022. Informed consent was obtained from all participants prior to the interviews. 

#### 2.2.2. Utility Elicitation

Eligible healthcare professionals who consented to participate were asked to complete a demographic questionnaire and then read the descriptions of the four hypothetical scenarios (None, HA/HA, HA/CI, and CI/CI). The interview was conducted either face-to-face or online via webcam by a well-trained interviewer. The interview consisted of two parts: the VAS and the TTO. For the VAS, firstly, the respondents were provided with five cards representing the four assessed health states and the “dead” state. They were asked to place each card, according to their preferences, along the VAS scale, which was anchored using the worst imaginable health state as 0 and the best imaginable health state as 100, with a higher score indicating higher desirability for the health state. The intervals or spacing between placements corresponded to the differences in preference as perceived by the respondent [22]. After all five health states were given a score between 0 and 100, the interviewer confirmed the placements with respondents and the scores were recorded. After completing the VAS, the interviewer explained TTO to the respondent and carried out a TTO exercise before the formal TTO assessment to familiarize the respondents with the process and confirm their understanding. A TTO board, in the form of a PowerPoint slide, was used as a visual aid to improve the respondents’ comprehension of TTO procedures. In the exercise, respondents were asked to imagine that they or their children were a child with allergic dermatitis and had ten years left to live. They were asked to choose between “live for ten years with allergic dermatitis” and “live for ten years in perfect health.” The interview would continue only if the latter was chosen, indicating the respondent’s correct understanding. Next, the respondents were asked to choose between “live for ten years with allergic dermatitis” and “die immediately”, and the length of life continued to change from 9.5, 0.5, 9.0, 1.0, etc., until the respondents perceived the two options equally preferable or when the answer was equal to the last one. Health utility values were elicited using this “ping-pong” method, which was an iterative procedure used to determine the indifferent point where high and low values were alternately presented [23,24]. After completing the exercise, the formal TTO interview was then conducted, repeating the procedure four times and each time replacing allergic dermatitis with one of the four hypothetical scenarios. 

### 2.3. Statistical Analysis

Respondents’ demographic characteristics were summarized via mean ± standard deviation (SD) for continuous variables and percentages for categorical variables. The calibration of the raw VAS scores was performed to transform the scores into utilities anchored at dead (=0) and perfect health (=1) [22] using the following formula:

(1) $$VAS-derived\;utility=\frac{\left(X-D\right)}{\left(100-D\right)}$$

*X*: the raw score of the assessed health state, *D*: the raw score of the “dead” state

In the TTO interview, for example, if the respondent was willing to trade off two of the ten offered years in order to regain perfect health, it was suggested that eight years in perfect health had the same value as ten years with the impaired health states. The respondent perceived living in the impaired health states at a value of 0.8 relative to perfect health (=1) [22]. Accordingly, the following formulas were used to calculate the TTO-derived utility values:

(2) $$TTO-derived\;utility=\frac{T}{10\;}$$

*T*: the indifferent point where the two options were equally preferable for the respondents.

Or

(3) $$TTO-derived\;utility=\frac{\left(T1+T2\right)/2}{10\;}$$

*T*1, *T*2: time, the last two points with equal answers, thus indicating preference reversal.

ANOVA, followed by Fisher’s Least Significant Difference (LSD) post hoc test, was used to find the differences in utility values among the four health states. All statistical analyses were conducted using IBM SPSS Statistics version 19. A *p*-value of <0.05 was used to determine statistical significance.

## 3. Results 

From October 2021 to March 2022, a total of 39 healthcare professionals were recruited from three medical centers, three regional hospitals, and one hearing rehabilitation institute for children. Two respondents did not complete the TTO interview, resulting in a total of 37 respondents being included in the analysis. The respondents’ demographic characteristics are presented in Table 1. The respondents included 27 women and 10 men, with a mean (SD) age of 39.6 (13.2) years and a mean (SD) working experience of 12.1 (9.9) years. A majority of the respondents were audiologists (64.9%) and working at medical centers (62.2%).

The utility scores of the four health states assessed are summarized in Table 2. The mean utility scores obtained from the VAS were 0.31 for no assistive devices, 0.41 for bilateral HAs, 0.63 for bimodal hearing, and 0.82 for bilateral CIs. In addition, the mean utility scores obtained from the TTO were 0.60 for no assistive devices, 0.69 for bilateral HAs, 0.81 for bimodal hearing, and 0.90 for bilateral CIs. In both the VAS and TTO methods, the utility values increased from the lowest values for no assistive devices to the highest values for bilateral CIs. The ANOVA result showed that the four groups did not all have the same VAS- or TTO-obtained utility values (*p* < 0.001). In addition, the results of the post hoc LSD pairwise comparisons showed that the difference was significant between any two groups (all *p* < 0.05).

## 4. Discussion 

Despite the significant economic and quality of life-related impacts of CIs in children, few studies have been conducted in this field. As pharmacoeconomic research in Asia expands into new areas, collecting local health utility data is essential for economic evaluations. This study is one of the few in Asia that used TTO to measure the health utilities for bilateral severe hearing impairment with different assistive devices. The utility values elicited in this study enhance the understanding of the relative impact of these hearing-assistive devices. Moreover, the values can be used in future CUA that can inform decision-makers about healthcare resource allocation.

Common measures for valuing health states include indirect questionnaire-based measures, such as the EuroQol five-dimensional questionnaire (EQ-5D), and direct valuation methods, such as standard gamble (SG) and TTO. Despite the convenience and popularity of the EQ-5D, however, it may not be appropriate for conditions affecting sensory functions, such as vision or hearing [25,26,27]. TTO is recommended by the NICE Guide as the measurement method in economic evaluation studies, and it is widely used for utility measurement. Moreover, compared to SG, which is cognitively demanding and time consuming, TTO is easy to administer. The present study demonstrated that the ping-pong method was feasible for the respondents in Taiwan. Nevertheless, the ways in which TTO with a ping-pong approach needs to be adapted in children with hearing loss and their parents need to be further examined. Given the cognitive challenges of the TTO, proxy-reported health utilities are an alternative way for valuing preferences in children [28,29]. Healthcare professionals can be proper proxy respondents if they are familiar with the health states assessed and have experience taking care of the studied patient population (e.g., the otolaryngologists and audiologists in the present study). 

The utilities elicited in the present study via VAS and TTO were both lowest for the health state without any assistive devices, followed by HA/HA, HA/CI, and CI/CI. The observation that the greatest quality of life is associated with CI/CI is consistent with previous studies [4,5,8,9,10,11], which indicates the benefits of bilateral CIs in children who had poor outcomes with HAs. CIs directly stimulate the auditory nerves in the cochlea and, therefore, improve the ability to hear, with a downstream benefit of increased speech intelligibility. Bilateral CIs provide further advantages for binaural hearing, such as the head shadow effect and enabling better speech comprehension, particularly in the noise background.

It was previously reported that the utility values obtained via TTO are generally higher than VAS-derived scores [30], which is consistent with the results collated in this study. Among all four health states examined in this study, the results showed that the TTO-derived utility values were higher than the VAS-derived scores for the same health state. In addition, the differences in TTO-derived utility values between HAs and CIs observed in this study are comparable to those reported in previous studies [12,14]. In this study, the utility value gained from bilateral CIs compared to bimodal hearing was 0.09, whereas a healthcare professional survey by Summerfield et al. reported the difference to be 0.05 [14]. Moreover, the utility increases from unilateral CI to bilateral CIs in previous studies varied from 0.03 to 0.126 [12,13,14,31,32], and the utility gain obtained in this study fell within that range.

There are a few limitations to this study. Firstly, the sample size was relatively small, though the number of respondents needed for utility assessment studies was not determined and the number of participants enrolled in this study was close to that of previous studies [13,14]. Secondly, the utilities derived may deviate from those that would be elicited from patients because experts generally provide higher utility values than patients, a bias that is attributed to the experts’ positive experiences with disease status after medical intervention [33,34]. Just as patients can better cope with chronic illnesses over time, experts tend to view these poor health states more favorably than the general population. As such, the health utilities obtained from healthcare professionals may not be representative of societal perspectives. 

An important implication of this study is the contribution of the utility values obtained to CUA, which assists policymakers in making informed decisions regarding resource allocation within the healthcare system. Since CUA plays a crucial role in determining the optimal allocation of public resources, it is essential to consider the perspectives of the general population. The health utilities derived from healthcare professionals may not be representative of societal perspectives. Future research is needed to expand the study population or generate experience-based utilities for patients’ individual health states.

HAs and CIs demonstrated clinical benefits in children with severe hearing loss. Considering the long-term use and economic burden of these assistive devices, comparative evaluations are required to support health authorities’ policy decision making. The utility values obtained in the study provide critical data for future CUA and health technology assessment.

## 5. Conclusions 

This study used both VAS and TTO to measure the health utility values of bilateral severe-to-profound hearing loss with assistive devices. The results of this study showed that bilateral CIs had the greatest utility value, followed by bimodal hearing, bilateral HAs, and, finally, no use of any assistive devices. 

The elicited utility values can serve as essential data for the calculation of health outcome measures in future CUA research. CUA is a common analysis method in pharmacoeconomic evaluation that enables direct comparison of interventions in different areas. Since health utility is jointly affected by multiple dimensions, such as physiological function, psychological health, and social wellbeing, the health preferences of individuals with different cultural backgrounds can also vary. As such, local utility data are preferred for CUAs. Furthermore, the present study can help clinical experts clarify the extent to which bilateral assistive devices improve patients’ quality of life and hearing.

## Figures and Tables

**Table 1:** Demographic characteristics of participants (N = 37).

Characteristics	Mean	±SD
**Age, years**	39.6	±13.2
**Practice experience, years**	12.1	±9.9
	**n**	(%)
**Sex**		
Female	27	(73)
Male	10	(27)
**Worksite**		
Medical center	23	(62.2)
Hearing rehabilitation institute	7	(18.9)
Regional hospital	7	(18.9)
**Profession**		
Audiologist	24	(64.9)
Otolaryngologist	10	(27.0)
Auditory–verbal specialist	1	(2.7)
Psychologist	1	(2.7)
Social worker	1	(2.7)

**Table 2:** Cochlear-associated health utilities.

Valuation Method	Health State	Mean Value (95% Confidence Interval)	*p*
VAS	None	0.31 (0.26–0.36)	<0.001
	HA/HA	0.41 (0.35–0.47)	
	HA/CI	0.63 (0.58–0.68)	
	CI/CI	0.82 (0.79–0.85)	
TTO	None	0.60 (0.52–0.69)	<0.001
	HA/HA	0.69 (0.63–0.76)	
	HA/CI	0.81 (0.77–0.86)	
	CI/CI	0.90 (0.87–0.94)	

None = without any assistive devices; HA = hearing aid; CI = cochlear implant; VAS = Visual analogue scale; TTO = Time trade off.

## Data Availability

The datasets analyzed during this study are not publicly available due to privacy restrictions.
